# Reasons for inadequate asthma control in children: an important contribution from the “French 6 Cities Study”

**DOI:** 10.1186/2049-6958-7-23

**Published:** 2012-08-08

**Authors:** Giuliana Ferrante, Stefania La Grutta

**Affiliations:** 1 CNR Institute of Biomedicine and Clinical Immunology, Palermo, Italy

## 

Asthma represents the most common chronic illness in children [1] and an important clinical and public health problem. In fact, diagnosing and treating asthma in children still remain a challenge. There is evidence that children with asthmatic symptoms are often undiagnosed and undertreated [2]. Considering the prevalence of childhood asthma and its associated burden, it is mandatory to obtain an optimal control of the disease and improving outcomes for patients [3]. To achieve this goal, guidelines were published with indications about medication use, control of the environment and health education. Unfortunately, evidence exists that guidelines recommendations are often not applied within the clinical practice [4]. Therefore, asthma control, as recommended by guidelines, has been shown to be satisfactory in less than 30% of children [1].

Diagnosis and asthma treatment depend on a complex interplay among morbidity, physician practice and access to health care [2].

Relating to morbidity, the association between asthma control and atopic disease is well known. It has been demonstrated that atopic comorbidities, such as rhinitis and eczema, are related to more severe asthma [5] and that treatment of allergic disease may improve asthma control [1].

Undoubtedly, the clinical status and the severity level of the disease are fundamental aspects relevant to asthma control, even if children who suffer from severe asthma (more frequent exacerbations, sleep disturbances, a high number of school day missed, activity limitations) could not receive a proper diagnosis and treatment, mainly due to social deprivation. In this context the contact with a doctor, through a better knowledge of the disease, could help the child with asthma to self-manage his condition. The problem of perception of symptoms by the patient and the family is strictly related. Patient’s and parent’s ability to recognize asthma symptoms depend on a patient-physician partnership. Some educational programs have been shown as useful in reducing asthma morbidity in children [6]. Interventions should also take into account the environment that is an important factor relevant to asthma pathogenesis and control. It is well known that environmental triggers such as outdoor and indoor allergens, passive smoking and particulate matter can elicit and exacerbate acute attacks in asthmatic children. In particular, passive exposure to parental tobacco smoke is a risk factor for childhood wheeze [2] and is associated with poor asthma control in children [1]. Moreover, it has been demonstrated that proximity to traffic (living near heavily polluted roadways or bus stop) has a negative impact on respiratory health of children, with increased risk of wheezing, medication use and diminished lung function [3,7].

The most recent GINA guidelines underline the physician’s role in asthma management and care, emphasizing that a proper control of the disease depends on doctor’s ability and experience in recognizing symptoms (considering possible differential diagnoses), defining the severity level (also by evaluating the respiratory function, as recommended by international guidelines), prescribing the correct medication and educating the patient and his family [6]. Recent data demonstrate that physicians often ignore guidelines [4] and the importance of using asthma control tools [1].

Finally, significant disparities in health care based on patient’s insurance status, education level, income and race/ethnicity are relevant to asthma under-diagnosis and under-treatment. Some reports from Europe [1] and North America [2] show that over 50% of children with asthmatic symptoms don’t receive treatment according to guidelines [3] and are more likely to be hospitalized or visited in the emergency department [2].

A new study by Annesi-Maesano et al. [8] published in this issue of *Multidisciplinary Respiratory Medicine* added new evidence to this field of research. Using data from the “French 6 Cities Study*”* conducted on a sample of 7.798 schoolchildren, aged 9–10 yr, living in metropolitan France, the Authors identified the main risk factors associated with the presence or absence of asthma diagnosis and treatment. In particular, they considered individual, socio-demographic, clinical and environmental factors. Children underwent clinical tests while their parents completed a standardized medical questionnaire. The population-sample studied comprised 903 asthmatic children: 58% had a doctor diagnosis, only of them 67% were treated for their condition.

The evaluation of the clinical condition showed some interesting evidences. First, the asthma severity level (evaluated according to GINA guidelines) was one of the main factors that influenced diagnosis and treatment. Most of undiagnosed and untreated children were in GINA level 1. Furthermore, in line with other studies [9], treatment was related to more severe asthma (more frequent exacerbations, sleep disturbances, hospitalizations, a high number of school days missed, activity limitations).Second, according to previous observations [10], diagnosed and treated asthmatic children had more allergic concomitant diseases, such as eczema and rhinitis. Therefore, co-morbidities and asthma severity seem to increase the likelihood of treatment.

Relevant to under-treatment of asthma, the Authors also highlighted inconsistencies about the type of treatment used. In fact, most of the treated children used bronchodilators for both attacks prevention and therapy. Unexpectedly, in the sample of children without a doctor diagnosis of asthma, there was someone who took medications (bronchodilators or inhaled corticosteroids) to improve its respiratory symptoms. These observations may suggest both a poor adherence of physicians to guidelines within clinical practice [4], and a non-adherence of patients to the treatment plan. Previous studies reported that even patients with severe asthma do not follow the treatment properly. Therefore, it is necessary to improve physician’s compliance to guidelines within clinical practice through educational interventions that enrich their awareness about diagnostic tools and therapy. Furthermore, it is important to identify subjects non-adherent to treatment. Since the reasons for poor adherence may vary among patients, individualized interventions that improve patient’s compliance to therapy are strongly desirable [11].

In addition, Annesi-Maesano et al. [8] confirmed that a low socio-economic status still represents an important factor in asthma management and care, limiting the access to health care system and consequently the optimal control of the disease.

At last, the Authors focused on environmental factors that can affect asthma management, particularly the exposure to passive smoking and urban traffic. They found that undiagnosed children were more exposed to maternal smoking and traffic. Moreover, they found that urban traffic (living near a bus stop) was the only environmental factor treatment-related. Proximity of an asthmatic’s house to a bus stop was an indicator of asthma severity and likelihood of treatment. Confirming existing data [1,3,7], these observations underline the necessity of taking into account the physical and social environment within clinical practice to improve management and care of asthmatic children.

In summary, the study by Annesi-Maesano et al. [8] shows that childhood asthma is still under-diagnosed and under-treated in metropolitan France. The Authors, by identifying the clinical, social and environmental characteristics of undiagnosed and/or undertreated children, highlighted the main factors that can be associated with absence of asthma diagnosis and treatment. Since a poor asthma control can have detrimental effects on children’ health, similar studies are warranted to understand what interventions are necessary to achieve a better management of this disease.
